# Cognition As a Therapeutic Target in the Suicidal Patient Approach

**DOI:** 10.3389/fpsyt.2018.00031

**Published:** 2018-02-13

**Authors:** Antônio Geraldo da Silva, Leandro Fernandes Malloy-Diniz, Marina Saraiva Garcia, Carlos Guilherme Silva Figueiredo, Renata Nayara Figueiredo, Alexandre Paim Diaz, António Pacheco Palha

**Affiliations:** ^1^Faculty of Medicine, University of Porto, Porto, Portugal; ^2^Brazilian Society of Dual Pathology, Brasília, Brazil; ^3^Universidade Federal de Minas Gerais, Belo Horizonte Brazil; ^4^Molecular Medicine Department, Universidade Federal de Minas Gerais, Belo Horizonte, Brazil; ^5^Associação Brasileira de Psiquiatria, Rio de Janeiro, Brazil; ^6^Universidade do Sul de Santa Catarina, Palhoça, Brazil

**Keywords:** suicide, cognition, impulsivity, attention bias, therapeutic target cognition, cognitive deficits, behavioral interventions, cognitive interventions

## Abstract

The current considerations about completed suicides and suicide attempts in different cultures call the attention of professionals to this serious public health problem. Integrative approaches have shown that the confluence of multiple biological and social factors modulate various psychopathologies and dysfunctional behaviors, such as suicidal behavior. Considering the level of intermediate analysis, personality traits and cognitive functioning are also of great importance for understanding the suicide phenomenon. About cognitive factors, we can group them into cognitive schemas of reality interpretation and underlying cognitive processes. On the other hand, different types of primary cognitive alterations are related to suicidal behavior, especially those resulting from changes in frontostriatal circuits. Among such cognitive mechanisms can be highlighted the attentional bias for environmental cues related to suicide, impulsive behavior, verbal fluency deficits, non-adaptive decision-making, and reduced planning skills. Attentional bias consists in the effect of thoughts and emotions, frequently not conscious, about the perception of environmental stimuli. Suicidal ideation and hopelessness can make the patient unable to find alternative solutions to their problems other than suicide, biasing their attention to environmental cues related to such behavior. Recent research efforts are directed to assess the possible use of attention bias as a therapeutic target in patients presenting suicide behavior. The relationship between impulsivity and suicide has been largely investigated over the last decades, and there is still controversy about the theme. Although there is strong evidence linking impulsivity to suicide attempts. Effective interventions address to reduce impulsivity in clinical populations at higher risk for suicide could help in the prevention. Deficits in problem-solving ability also seem to be distorted in patients who attempt suicide. Understanding cognitive changes in patients who attempt suicide open an important perspective in the approach of patients with mental disorders. Identifying cognitive deficits in these patients, along with personality traits, depressive symptoms, and suicidal cognitive schemas may indicate to the psychiatrist the need for emergency care. Behavioral and cognitive interventions have been associated with reductions in suicide ideation, as well as suicide attempts in different populations.

The current considerations about complete suicides and suicide attempts in different cultures call the attention of professionals from different areas to this serious public health problem. It is estimated that almost 1 million people commit suicide per year worldwide. In Brazil, there are 32 complete suicides every day from which about 96.8% had some identifiable mental disorder.

Suicidology is the field of interdisciplinary knowledge that brings together professionals from different specialties to enable them to construct proposals of prevention and acute intervention from the integration of different levels of analysis. Considering the levels of analysis derived from the basic sciences, the search for biomarkers related to suicidal behavior points, for example, to the participation of neural systems associated to the modulation of responses to stressors (e.g., hypothalamic–pituitary–adrenal axis and the noradrenergic system of the locus coeruleus), neuroinflammatory mechanisms system (e.g., increased levels of interleukins 1 and 6 in the frontopolar cortex), and also the serotonergic hypofunction (1). On the other hand, considering the levels of macroscopic analysis studied by Sociology, the relationship between the individual and society has been considered since the first trials to understand the phenomenon. Durkheim’s classic works (2), for example, already predicted different types of suicide sociologically determined such as the anomic (suicidal behavior that happens because of the acute social chaos), fatalistic (suicide due to the lack of hope of overcoming oppression and of repression of individual freedom), altruistic (suicide motivated by an ideal that the subject considers him/herself as more important than his/her own life), and selfish (characterized by the chronicity of a weak bond between the person and the society).

More recently, integrative approaches have shown that the confluence of multiple biological and social factors modulate diverse psychopathologies and dysfunctional behaviors, such as suicidal behavior (3). Some of the results of the Dunedin longitudinal study clearly show that throughout life, biological vulnerability factors added to adverse social conditions increase the chance of ideation and suicide attempts (4). In this study, individuals who presented two copies of the short allele of this gene (related to a lower serotonergic function) and experienced more stressful situations presented a higher risk of showing ideation and suicide attempts. In this perspective, Brodsky (5), using the stress-diathesis model, suggests that adverse childhood experiences (e.g., family stress, abuse, and other types of violence) associated with biological characteristics (e.g., serotonergic hypofunction and HPA axis dysfunction) can create a vulnerability factor that, in adult life, in the face of stressful situations increases the risk of suicidal behavior. This, in its turn, involves self-aggression, suicidal preparation, the attempt and the act itself.

Considering the level of intermediate analysis, the one of individual differences, personality traits, and cognitive functioning, are also of great importance for understanding the suicidal phenomenon. For example, DeShong et al. (6) have demonstrated that personality traits can be considered important predictors of suicidal ideation. The results of this study indicate that high levels of neuroticism and low levels of extroversion are related to the current presence of suicidal ideation in a sample of university students. The authors also verified that high levels of neuroticism are positively and significantly correlated with two other personality traits that indicate the high risk for a suicide attempt: the perception of oneself as burdensomeness for the others (Perceived Burdensomeness) and the perception to be disconnected from society (Thwarted Belongingness). Moreover, the relationship between personality traits and the success of interventions has also been verified. For example, the extroversion trait seems to make the individual more sensitive to social support. In situations of low social support, extroversion tends to be significantly related to suicidal ideation (7).

## Cognition and Suicide Behavior

In relation to cognitive factors, we can group them into cognitive schemas of reality interpretation and basic cognitive processes. Cognitive schemata of hopelessness (belief that the situation will not be resolved in the future) and intolerance to suffering are examples of interpretation patterns of reality in patients with suicidal ideation (8). On the other hand, different types of primary cognitive alterations are related to suicidal behavior, especially those resulting from changes in frontostriatal circuits (9). Among such cognitive mechanisms can be highlighted the attentional bias for environmental cues related to suicide, impulsive behavior, verbal fluency deficits, non-adaptive decision-making, and reduced planning skills.

Attentional bias consists in the effect of thoughts and emotions, frequently not conscious, about the perception of environmental stimuli. Attentional bias makes the individual to pay too much attention to specific environmental cues that are related to the psychiatric disorder he/she presents (10). According to Wenzel et al. (8), suicidal ideation and hopelessness can make the patient unable to find alternative solutions to their problems other than suicide, biasing their attention to environmental cues related to such behavior.

One of the most used tasks to evaluate attentional bias is the modified Stroop paradigm, involving processing of stimuli related to emotion. Stroop paradigms of an emotional nature usually require the patient to say, as quickly as possible, the name of the color with which certain words were written. These words may be neutral or have negative or positive emotional valence. In studies of attentional bias for suicide, neutral words, positive valence words, words with general negative valence, and words with negative valence related to suicide are generally used. As the patient processes more automatically read the word and give the desirable answer, that is, to name the color. Richard-Devantoy et al. (11) verified that patients with a history of suicide attempt perform poorly on emotional Stroop tasks when they have to process suicide-related words. Cha et al. (12), in a longitudinal study, found that the attentional bias for suicide-related stimuli is an important predictor of future attempts. In this study, the authors evaluated patients treated in the psychiatric emergency with a modified version of the Stroop test containing words with positive (e.g., happiness), negative (e.g., solitude), neutral valence (e.g., museum), and suicide related (e.g., death). Besides presenting a greater attention bias for words related to suicide, attentional bias was an important predictor of further attempts during the 6 months after the evaluation.

Bias in the processing of emotional information may also influence the way facial expressions are identified, even in individuals with subclinical depressive symptoms who have suicidal ideation. Maniglio et al. (13) verified, in a sample of subjects of the general population, that the presence of suicidal thoughts was related to the tendency to interpret neutral faces as they were expressing sadness.

Recent research efforts are directed to assess the possible use of attention bias as a therapeutic target in patients presenting suicide behavior. In a randomized control trial (RCT), a community sample of individuals who reported past month suicide ideation and inpatients admitted for suicide ideation or attempt were submitted to four training sessions of attentional bias modification (ABM) and their cognitive performance compared to a control group (14). The authors did not find significant differences between the groups even stratifying the analysis for severe suicide ideators. Possible reasons for the negative findings included number and duration of training sessions, lack of AMB efficacy for suicide-specific attentional bias, and type II error due to sample size (14). Other studies addressed the potential of AMB in alleviating clinical conditions that are known risk factors for suicide attempts. In a trial for testing the potential of ABM in reducing depressive symptoms in adolescents with depressive disorders, a major risk for suicidal behaviors (15), Yang et al. have compared active AMB intervention versus placebo ABM training (16). The former was associated with reduction on clinical-rated and self-reported depressive symptoms compared with the control group at 12-month follow-up assessment, as well as with higher remission rates (16). On the other hand, de Voogd et al., throughout a multi-center RCT with a non-clinical sample of adolescents, did not find significant differences regarding anxiety and depressive symptoms between intervention and control group (17). Insomnia, another clinical characteristic associated with suicidal behaviors (18, 19), was a target for ABM in a RCT by Lancee et al. (20). The results showed no evidence of efficacy for AMB in decreasing sleep symptoms problems, which may be related to the low level of attention bias at baseline (20). The discrepancy in the literature for the AMB as a potential therapeutic tool for treating suicidal thoughts as well as its risk factors, suggests that further studies exploring different protocols interventions and clinical sample characteristics should be performed.

The relationship between impulsivity and suicide has been largely investigated over the last decades and there is still controversy about the theme. Although there is strong evidence linking impulsivity to suicide attempts, and particularly to violent attempts [see, for example, Ref. (21)], Smith et al. (22) suggest that, if suicide is planned, the suicide behavior could not, in these cases, be explained by the impulsive tendency. Reyes-Tovilla et al. (23) verified important differences between people who attempt suicide in an inapposite way and those who premeditate the attempt. The second group tends to try in a more lethal way, has higher rates of comorbidity with alcoholism, and use cannabis besides having lower level of education. On the other hand, given the multidimensional nature of impulsivity, it is plausible to think that some specific types of impulsive manifestation would be more related to suicidal behavior. Thus, Malloy-Diniz et al. (24) and Neves et al. (25) verified that the impulsive and immediate decision-making is more related to the suicide attempts in bipolar patients. It is plausible to think here that even planned suicides could be understood by these results, yet planning would focus on the end of immediate suffering. These findings have been consistently replicated by other studies (11) showing that the decisional focus on psychiatric patients may be one of the risk factors for the suicide act.

Impulsivity can serve both as a moderator and mediator variable in the association between several diagnostic and clinical conditions with suicide ideation or attempts. Wang et al. (26) evaluated 162 patients with major depressive disorder and found that those with higher impulsivity, regardless depression severity, were more likely to present suicide ideation (26). In addition, specific neural circuits associated with impulsivity and aggression may mediate the lethality of suicidal behavior in patients with borderline personality disorder (27).

Thus, effective interventions address to reduce impulsivity in clinical populations at higher risk for suicide could help in the prevention. An internet-based psychoeducation approach was compared to a control group with no psychoeducation for the treatment of women with DSM-IV (28) criteria for borderline personality disorder. Among several others outcomes, the experimental group with psychoeducation showed significantly decrease in impulsivity scores (29). Thylstrup et al. (30) applied a different type of psychoeducational program for patients with substance use and antisocial personality disorder in a randomized trial. The Impulsive Lifestyle Counselling, with sessions that included linking “patients’ impulsive behaviors to the immediate consequences,” was associated with positive effects in reducing substance use behaviors, a major suicide risk factor (30, 31). Impulsivity was also decreased in a sample of high school students, an age group which suicide is the second main cause of dead (32), after a mindfulness training program (MTP). Ten weekly sessions of the MTP was associated with significantly reduction in cognitive, motor, and non-planning dimensions of impulsivity as compared to the control group (33).

As mentioned above, impulsive decision-making related to imediatistic focus regardless long-term consequences is frequently associated with suicide behavior. Oldershaw et al. (34) evaluated whether there was an association between improvements in decision-making and reduction on suicide ideation in adolescents with a history of self-harm, after a CBT treatment (34). According to the authors, the therapy has included elements with the aim of strength decision-making skills, measured in this study by the Iowa Gambling Task (IGT) (35). Despite improvements of IGT scores within the CBT group, changes in IGT scores did not correlate with suicide ideation (34).

Deficits in problem-solving ability also seem to be distorted in patients who attempt suicide. In particular, in situations of stress, problem-solving skills are important factors in minimizing the effect on mental health. In this perspective, Grover et al. (36) verified that deficits in problem-solving abilities in patients submitted to moderate and high situations of stress are related to suicidal behavior in adolescents. Khan et al. (37) found that university students who present productive strategies to manage stressful situations (work with the focus on the problem, maintaining optimism, seeking help and support from other people) and social support had lower chances to attempt suicide. In a study that evaluated the relationship between problem-solving skills and suicidal ideation in people who experienced childhood abuse, Kwok et al. (38) found that the rational style of problem-solving acts as a moderator in the relationship between abuse in childhood and suicide attempt in adulthood. Such association was described only in the women who participated in the study. The style of problem solving also seems to be related to suicidal behavior. Quinones et al. (39) found that patients who attempt suicide tend to present more passive strategies of solution of problems, that is, they are dependent on the action of other people, related to luck and chance or over time.

Cognitive-behavioral problem solving was compared to “treatment as usual” for several outcomes, including suicide ideation and attempts, in a sample of high-risk individuals, with positive findings, especially in short term (40). Cognitive therapy was also associated with a faster improvement in negative problem orientation among individuals with history of recent suicide attempt in another randomized controlled trial (41). Problem-solving therapy may be especially effective in older patients with depression and executive dysfunction. Gustavson et al. (42) reported lower frequency of reported suicide ideation in the experimental group in comparison to supportive therapy up to 36 weeks after treatment (42). At least part of the effectiveness of mindfulness-based interventions for suicide prevention has also been related by means of improvement in problem solving, as well as attentional dyscontrol and abnormal stress response (43). Thus, therapies with the aim of improve problem-solving abilities, especially active problem solving (39), should be considered in individuals at higher risk for suicide.

## Conclusion

Evidences linking cognitive deficits and suicide behavior are consistent. Jollant et al. (9) group the main cognitive difficulties in people who attempt suicide in three categories that synthesize the above-mentioned findings and aggregate other described changes. The categories include

(1) Changes in the modulation and attribution of values to the experiences, which would involve an attentional and emotional biased response to environmental stimuli and less adaptive decisions in relation to the perception of environmental risks.
(2) Deficits in emotional and cognitive regulation including cognitive inflexibility, poor repertoire related to planning and problem solving, lowered verbal fluency affecting communicational ability.
(3) Behavioral facilitation in emotional contexts, characterized by impulsive response in situations of major stress and affective overload.

Moreover, cognitive deficits in psychiatric patients are important therapeutic targets, despite the paucity of intervention studies considering cognition as a therapeutic target in suicide patients, training self-regulatory processes, including decision-making skills, efficient problem solving, and impulse control present potential for clinical use in suicide prevention.

Behavioral and cognitive interventions has been associated with reductions on suicide ideation, as well as suicide attempts in different populations (1–4), probably by targeting different cognitive dysfunctions associated to suicide behaviors, in addition to anxiety and depressive symptoms. Thus, specific interventions toward these cognitive domains, such as attentional bias, impulsivity, problem solving, and decision-making, could help to maximize the efficacy of the available therapeutic options. Future studies are needed to evaluate the effectiveness of cognitive training for this purpose.

## Author Contributions

All authors listed have made substantial, direct, and intellectual contribution to the work and approved it for publication.

## Conflict of Interest Statement

The authors declare that the research was conducted in the absence of any commercial or financial relationships that could be construed as a potential conflict of interest.
